# Intercepting biological messages: Antibacterial molecules targeting nucleic acids during interbacterial conflicts

**DOI:** 10.1590/1678-4685-GMB-2022-0266

**Published:** 2023-03-06

**Authors:** Julia Takuno Hespanhol, Lior Karman, Daniel Enrique Sanchez-Limache, Ethel Bayer-Santos

**Affiliations:** 1 Universidade de São Paulo, Instituto de Ciências Biomédicas, Departamento de Microbiologia, São Paulo, SP, Brazil.

**Keywords:** Antibiotics, bacteriocins, effectors, DNase, RNase

## Abstract

Bacteria live in polymicrobial communities and constantly compete for resources. These organisms have evolved an array of antibacterial weapons to inhibit the growth or kill competitors. The arsenal comprises antibiotics, bacteriocins, and contact-dependent effectors that are either secreted in the medium or directly translocated into target cells. During bacterial antagonistic encounters, several cellular components important for life become a weak spot prone to an attack. Nucleic acids and the machinery responsible for their synthesis are well conserved across the tree of life. These molecules are part of the information flow in the central dogma of molecular biology and mediate long- and short-term storage for genetic information. The aim of this review is to summarize the diversity of antibacterial molecules that target nucleic acids during antagonistic interbacterial encounters and discuss their potential to promote the emergence antibiotic resistance.

## Introduction

Bacteria live in dense polymicrobial communities constantly competing for resources and use either exploitative competition in which molecules like siderophores can be used to improve the acquisition of micronutrients; or interference competition in which cytotoxic molecules are used to inactive target cells (Granato *et al*., 2019). During evolution, bacteria have evolved a diverse array of weapons to inhibit the growth or kill competitors, which are broadly divided into contact-independent and contact-dependent antagonistic mechanisms (Peterson *et al*., 2020). These weapons specialized for biological conflicts evolved to target many cellular components essential for life, such as the genetic information flow through the central dogma, the cell wall, membranes, and key molecules like NAD^+^. As bacteria have been fighting these microscopic battles for millions of years using diverse antimicrobial molecules, it is not a surprise that studies on bacteria preserved in frozen glaciers identified the presence of antibiotic resistance genes that pre-dated human discovery of the first antibiotic (Mindlin and Petrova, 2017). In this review, we will examine molecules such as antibiotics, bacteriocins and effectors produced by bacteria and used during interbacterial conflicts to target DNA and several types of RNAs. We will end by highlighting the underappreciated but important role of these molecules in promoting antimicrobial resistance in natural environments.

## Molecules of the central dogma

In molecular biology, the central dogma is an explanation of the flow of genetic information within a biological system. It refers to the information passing from DNA to RNA, and RNA to proteins (Crick, 1970; Morange, 2009). The machinery associated with their synthesis are among the most conserved and (arguably) important molecules within a living cell. DNA and RNA are polymers of nucleotides, which are composed of a nitrogenous base, a pentose sugar, and a phosphate group (Rich, 1959; Minchin and Lodge, 2019). The bases are either purines (adenine or guanine), or pyrimidines (cytosine and thymine for DNA or uracil for RNA). The nucleotides are connected by phosphodiester bonds between the 5’-phosphate group and the 3’-hydroxyl group, while the bases adenine/thymine (or adenine/uracil for RNA) and guanine/cytosine establish hydrogen bonds (Rich, 1959; Minchin and Lodge, 2019).

DNA replication occurs in a semiconservative manner (Meselson and Stahl, 1958; Hanawalt, 2004). Helicases use energy of ATP hydrolysis to open the double-strand (Abdel-Monem *et al*., 1976; Oakley, 2019), DNA primases synthesizes RNA primers that will be used by DNA polymerases (Scherzinger *et al*., 1977; Oakley, 2019), while topoisomerases help in the unwinding process (Wang, 1971). Preservation of the integrity of the genomic information is fundamental for life and there are many DNA repair mechanisms that can either correct errors originated during replication or fix damages induced by external agents (Schärer, 2003). Damaged nucleotides can be repaired by base excision repair (BER) or nucleotide excision repair (NER) (Uphoff and Sherratt, 2017). BER recognizes abnormal bases in the nucleotides along the DNA molecule, such as uracil that spawn from cytosine deamination. BER includes the hydrolyzation of the abnormal base from the nucleotide, followed by the cleavage of the DNA by endonucleases (Uphoff and Sherratt, 2017). Meanwhile, NER removes an entire nucleotide that causes large distortions in the DNA double-helix, and includes the recognition of the lesion by the enzymes UvrA and UvrB, followed by incision at flanking sites of the distortion by UvrC endonuclease and displacement of the damaged strand by UvrD helicase (Uphoff and Sherratt, 2017). After excision from both BER or NER, DNA polymerase I and DNA ligase resynthesize DNA in the gap (Uphoff and Sherratt, 2017). A double-strand break (DSB) can be repaired by homologous recombination (HR) that preserves the previous genetic information or by non-homologous end-joining (NHEJ), which can lead to the loss or alteration of the original information (Wyman *et al*., 2004; Shuman and Glickman, 2007).

The information stored in DNA is decoded into RNAs by RNA polymerases (RNAP) (Ebright, 2000). The transcribed RNA could be a transfer RNA (tRNA), a ribosomal RNA (rRNA) or a messenger RNA (mRNA). In bacteria, the 70S ribosome is composed by two subunits: the 30S subunit comprises the 16S rRNA and 21 proteins; while the 50S subunit contains the 23S rRNA, 5S rRNA and 33 proteins (Deutscher, 2009). In several cases, the final step in the expression of the information contained in genes is the synthesis of proteins (Rodnina, 2018), which begins with the association of the ribosome with an mRNA via interaction of the 30S subunit with the Shine-Dalgarno sequence in the mRNA (Shine and Dalgarno, 1974). The elongation follows as the codon in the mRNA is exposed to match the corresponding anti-codon of an aminoacyl-tRNA. The peptidyl transferase center of the ribosome establishes the peptide bond, which is mediated by a catalytic rRNA (Monro, 1967). Overall, fidelity and effectiveness of these steps are required for the maintenance of genetic information and its transfer into molecules that perform work inside living cells.

## Bacterial antagonistic mechanisms

**Figure 1 -:**
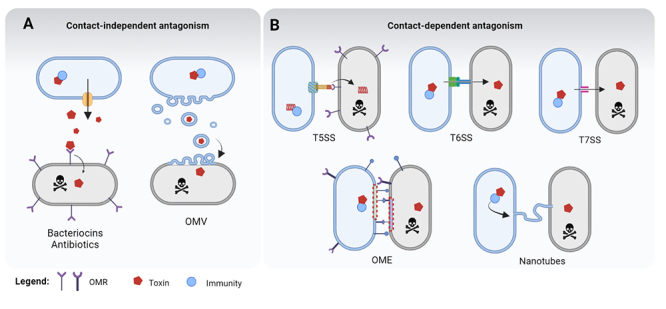
Antagonistic strategies used by bacteria to counteract competitors. (A) Contact-independent antagonism. Colicins, microcins and antibiotics (red hexagon) reach targets by binding to OMRs (outer membrane receptors) prior to internalization. Autointoxication is prevented by immunity proteins, degrading/modifying proteins or efflux pumps (blue circles). Outer membrane vesicles (OMVs) deliver toxins to competing bacteria by membrane fusion. **(B)** Contact-dependent antagonism. T5SS presents CdiB anchored in cell membrane and CdiA extended. Receptor-binding domain (RBD) of CdiA interacts with OMR of targets to translocate CdiA-CT (red) into competitors. T6SS is anchored in the cell membrane and upon contraction propelled into target cell to deliver toxins (red hexagon). T7SS effectors (red hexagons) secreted into target cells upon contact. Outer membrane exchange (OME) events can transfer toxic proteins (red hexagons) that reach targets. Nanotubes are membrane extensions that connect two bacteria to transport toxins (red hexagons). Cognate immunity proteins produced by attacking bacteria are represented by blue circles. Created with BioRender.com.

Bacteria inhabit complex environments where they interact and compete with other organisms, both prokaryotic and eukaryotic. Several systems specialized in biological conflict, both defensive and offensive, emerged during evolution to combat competitors, predators, and parasites (Figure 1). These systems participate in an arms race in which their genes have a high rate of evolution. Probably the most well-known antibacterial molecules are antibiotics, which are produced by a variety of organisms (Berdy, 2005). Antibiotics are bioactive secondary metabolites not synthesized by ribosomes (Berdy, 2005). They belong to different classes, usually based on their molecular strutures, and target several metabolic processes, including those related to the central dogma (Etebu and Arikekpar, 2016). These molecules are produced and secreted in the extracellular environment by ATP-binding cassette (ABC) transporters (Méndez and Salas, 2001). Producing-bacteria are protected from antibiotics by different mechanisms, including the synthesis of efflux pumps or specific enzymes that degrade/modify the antibiotic or its target (Darby *et al*., 2022) (Figure 1).

Bacteriocins are another type of biomolecule used in antagonistic encounters that are synthesized by ribosomes and can be divided into colicins and microcins (Cascales *et al*., 2007). Colicins are larger bacteriocins (>10kDa) secreted by a diversity of bacteria, and *Escherichia coli* was the first and most extensively studied. Colicins have three domains: an N-terminal translocation domain, a central receptor-binding domain and a toxic C-terminal domain (Cascales *et al*., 2007). These proteins are released in the medium and are internalized by binding to specific outer membrane receptors. Colicin-producers encode immunity proteins that bind to the toxic domains to neutralize their effect (Cascales *et al*., 2007). The expression of these proteins is largely regulated by the SOS response to DNA damage (Walker, 1996; Cascales *et al*., 2007). Microcins consist of smaller polypeptides (<10kDa) that require post-translational modification prior to secretion. Microcins target closely related species via binding to outer membrane receptors, and immunity is conferred either by a specific protein that interacts with the microcin or by efflux pumps (Duquesne *et al*., 2007) (Figure 1). 

Many types of macromolecular complexes, named protein secretion systems, are key players in bacterial antagonist interactions (Klein *et al*., 2020). These include the T1SS, T4SS, T5SS and T6SS of Gram-negative bacteria and T7SS of Gram-positives (Figure 1) (Klein *et al*., 2020). The T1SS uses glycine-zipper proteins that form large aggregates in the producer outer membrane and kill target bacteria upon contact (García-Bayona *et al*., 2017). The bacteria killing T4SS apparatus is evolutionarily related to the conjugative machinery and relays on the coupling protein VirD4 for effector selection and translocation into competitors through an extracellular pilus (Souza *et al*., 2015). A subtype of T5SS mediating contact-dependent growth inhibition (CDI) is composed of two proteins, an outer membrane protein CdiB that anchors an exoprotein with a central receptor-binding and a C-terminal toxic domain (CdiA), which interacts with an outer membrane receptor at a target cell to deliver the toxic C-terminus (Aoki *et al*., 2005). The T6SS is a contractile nanomachine evolutionarily related to bacteriophage tails that fire an array of effectors inside target cells at each contraction event (Hood *et al*., 2010; Basler, 2015). The T7SS secretes effectors with an LXG N-terminal and C-terminal toxic domains and participates in bacterial competition in Gram-positives (Cao *et al*., 2016). The vast array of macromolecules specialized in interbacterial conflicts reinforce their importance for bacterial fitness. 

The protein complexes described above only mediate the secretion/translocation of the real key players in bacterial antagonism: the toxic molecules used to poison targets cells. In bacteria, there are two main types of toxic molecules: proteinaceous and small molecules (Ruhe *et al*., 2020). Proteinaceous antimicrobials contemplate ribosome-synthesized molecules, such as bacteriocins and effectors (Ruhe *et al*., 2020), while antibiotics are synthetized via the secondary metabolism (Walsh, 2016). Many effector proteins contain multiple domains, usually a conserved N-terminus that engage in protein export that varies according to the secretion system it is associated with (Ruhe *et al*., 2020); and a variable C-terminus that contains the toxic domains (Zhang *et al*., 2012; Ruhe *et al*., 2020). Effectors with this configuration are commonly known as polymorphic toxins (Zhang *et al*., 2012; Ruhe *et al*., 2020). Next, we will discuss these two main types of antibacterial molecules.

## Proteinaceous Antimicrobials Targeting Nucleic Acids

**Figure 2 -:**
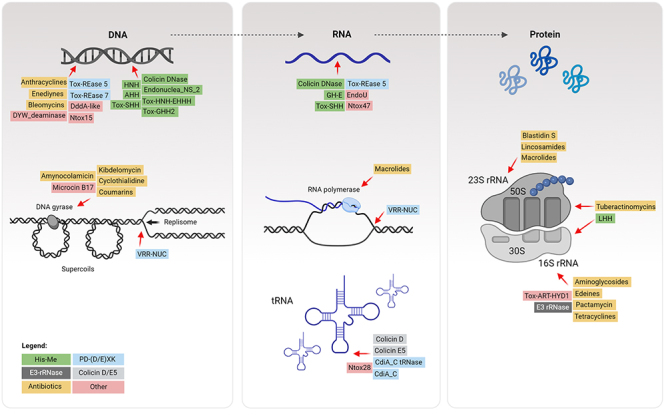
Antibiotics, bacteriocins, and effectors targeting nucleic acids. Schematic representation of the information flow through the molecules of the central dogma (DNA, RNA and protein). Antibiotics, bacteriocins, and contact-dependent effectors targeting nucleic acids either by binding and inhibition or by enzymatic cleavage are indicated. Molecules were grouped according to their protein domains: His-Me finger (green), PD-(D/E)xK (blue), Colicin D/E5 (light grey), E3-rRNAse (dark grey), antibiotics (orange), others (light red). The complete list of molecules is described in Table 1. Created with BioRender.com.

A large variety of DNase and RNase domains have been predicted by *in silico* analysis of polymorphic toxins (Zhang *et al*., 2012). Most DNase effectors experimentally characterized to date belong to the His-Me finger superfamily (Pfam CL0263) or to the PD-(D/E)xK superfamily (CL0236). On the other hand, RNase effectors are more diverse and belong to the colicin D/E5 (CL0640), Ntox28 (PF15605), EndoU (CL0695) and PD-(D/E)xK (Table 1, Figure 2).

**Table 1:** - Antibacterial molecules that target nucleic acids.

Name	Activity	Classification (Pfam)		Organism	References
		Superfamily	Family/Clade		
Bacteriocin					
Carocin D	DNase	His-Me_finger (CL0263)	-	*Pectobacterium carotovorum*	Roh *et al*., 2010
Carocin S1	DNase	-	-	*Pectobacterium carotovorum*	Chuang *et al*., 2007
Carocin S3	DNase	-	-	*Pectobacterium carotovorum*	Wang *et al*., 2020
Colicin E2	DNase	His-Me_finger (CL0263)	Colicin DNase (PF12639)	*Escherichia coli W3110*	Schaller and Nomura, 1976
Colicin E7	DNase RNase	His-Me_finger (CL0263)	Colicin DNase (PF12639)	*Escherichia coli K317*	Males and Stocker, 1980; Chak *et al.*, 1991; Hsia *et al.,* 2004
Colicin E8	DNase	His-Me_finger (CL0263)	Colicin DNase (PF12639)	*Escherichia coli J*	Cooper and James 1984; Toba *et al.*, 1988
Colicin E9	DNase RNase	His-Me_finger (CL0263)	Colicin DNase (PF12639)	*Escherichia coli J*	Cooper and James 1984; Chak *et al.,* 1991; Garinot-Schneider *et al*., 1996; Pommer *et al.,* 1998
Klebicin A	DNase	His-Me_finger (CL0263)	Colicin DNase (PF12639)	*Klebsiella pneumoniae*	Cooper and James, 1985; James *et al*., 1987
Klebicin B	DNase	His-Me_finger (CL0263)	Colicin DNase (PF12639)	*Klebsiella pneumoniae*	Riley *et al*., 2001
Pyocin AP41	DNase	His-Me_finger (CL0263)	Colicin DNase (PF12639)	*Pseudomonas aeruginosa*	Sano and Kageyama, 1981; Sano *et al.,* 1993
Pyocin S1	DNase	His-Me_finger (CL0263)	Colicin DNase (PF12639)	*Pseudomonas aeruginosa*	Sano *et al*., 1993
Pyocin S2	DNase	His-Me_finger (CL0263)	Colicin DNase (PF12639)	*Pseudomonas aeruginosa*	Ohkawa *et al.*, 1973; Sano *et al.*, 1993
Pyocin S3	DNase	-	-	*Pseudomonas aeruginosa*	Duport *et al.,* 1995
Pyocin S8	DNase	His-Me_finger (CL0263)	Colicin DNase (PF12639)	*Pseudomonas aeruginosa*	Turano *et al*., 2017; Turano *et al.*, 2020
Pyocin S9	DNase	His-Me_finger (CL0263)	Colicin DNase (PF12639)	*Pseudomonas aeruginosa*	Ghequire and De Mot, 2014
Usp	DNase	His-Me_finger (CL0263)	Colicin DNase (PF12639)	*Escherichia coli*	Kurazono, 2000; Nipic *et al*., 2013; Zaw *et al.,* 2013
Carocin S2	tRNase	Colicin D/E5 (CL0640)	Colicin_D (PF11429)	*Pectobacterium carotovorum*	Chan *et al*., 2011
Colicin E5	tRNase	Colicin D/E5 (CL0640)	Colicin_E5 (PF12106)	*Shigella sonnei 101BM*	Males and Stocker, 1982; Ogawa *et al.,* 1999; Masaki and Ogawa, 2002
Colicin D	tRNase	Colicin D/E5 (CL0640)	Colicin_D (PF11429)	*Escherichia coli K-12 W1485*	Timmis and Hedges, 1972; Tomita *et al*., 2000; Masaki and Ogawa, 2002
Klebicin D	tRNase	Colicin D/E5 (CL0640)	Colicin_D (PF11429)	*Klebsiella pneumoniae*	Chavan *et al*., 2005
Pyocin S4	tRNase	Colicin D/E5 (CL0640)	Colicin_E5 (PF12106)	*Pseudomonas aeruginosa*	Parret and De Mot, 2000
Pyocin S6	rRNase	-	E3 rRNase (PF09000)	*Pseudomonas aeruginosa*	Dingermans *et al*., 2016
Cloacin DF13	rRNase	-	E3 rRNase (PF09000)	*Enterobacter cloacae*	De Graaf *et al*., 1973
Colicin E3	rRNase	-	E3 rRNase (PF09000)	*Escherichia coli CA38 Pseudomonas spp.*	Senior and Holland, 1971; Bowman *et al*., 1971; Lasater *et al.,* 1989 *;* Ogawa *et al.*, 1999
Colicin E4	rRNase	-	E3 rRNase (PF09000)	*Citrobacter 20-78*	Horak, 1975; Smarda *et al.,* 1988; Smarda *et al.,* 2002; Hirao *et al.,* 2004
Colicin E6	rRNase	-	E3 rRNase (PF09000)	*Shigella sonnei*	Males and Stocker, 1982; Sharma *et al.,* 2002; Hirao *et al.,* 2004
Klebicin C	rRNase	-	E3 rRNase (PF09000)	*Klebsiella pneumoniae*	Chavan *et al*., 2005
Microcin B17	DNA gyrase	-	-	*Escherichia coli Pseudomonas spp.*	Baquero and Moreno, 1984; Moreno and Baquero 1986; Heddle *et al.,* 2001
**T4SS**					
Smlt4382	DNAse	His-Me_finger (CL0263)	AHH (PF14412)	*Stenotrophomonas maltophilia*	Bayer-Santos *et al.,* 2019
XAC3266	DNAse	His-Me_finger (CL0263)	AHH (PF14412)	*Xanthomonas citri*	Souza *et al.,* 2015
**T5SS**					
CdiA-CT^3937-2^	DNase	-	-	*Dickeya dadantii*	Aoki *et al*., 2010
CdiA_2_-CT	DNase	PD-(D/E)XK (CL0236)	Tox-REase 7 (PF15649)	*Acinetobacter baumannii*	Roussin *et al*., 2019
CdiA-CT^GN05224^	RNase	EndoU (CL0695)	EndoU_bacteria (PF14436)	*Klebsiella aerogenes GN05224*	Michalska et al., 2018
CdiA-CT^STECO31^	tRNase	EndoU (CL0695)	EndoU_bacteria (PF14436)	*Escherichia coli STEC_O31*	Michalska et al., 2018
CdiA-CT_II_^Bp1026b^	tRNase	PD-(D/E)XK (CL0236)	CdiA_C (PF18451)	*Burkholderia pseudomallei*	Morse *et al*., 2012
CdiA-CT^E479^	tRNase	PD-(D/E)XK (CL0236)	CdiA_C_tRNase (PF18664)	*Burkholderia pseudomallei*	Nikolakakis *et al*., 2012
CdiA-CT^EC869^	tRNase	Colicin D/E5 (CL0640)	-	*Escherichia coli EC869*	Jones *et al*., 2017
CdiA-CT^EC3006^	tRNase	Colicin D/E5 (CL0640)	Colicin_D (PF11429)	*Escherichia coli EC3006*	Willett *et al*., 2015; Gucinski *et al*., 2019
CdiA-CT^Kp342^	tRNase	Colicin D/E5 (CL0640)	Colicin_D (PF11429)	*Klebsiella pneumoniae 342*	Gucinski *et al*., 2019
CdiA-CT^K96243^	tRNase	Colicin D/E5 (CL0640)	Colicin_E5 (PF12106)	*Burkholderia pseudomallei*	Nikolakakis *et al*., 2012
CdiA-CT^E478^	tRNase	Colicin D/E5 (CL0640)	Colicin_E5 (PF12106)	*Burkholderia pseudomallei*	Nikolakakis *et al*., 2012
CdiA-CT^UPEC536^	tRNase	-	Ntox28 (PF15605)	*Escherichia coli UPEC536*	Aoki *et al*., 2010; Diner *et al.*, 2012
CdiA-CT_o1_^EC93^	tRNase	-	Ntox28 (PF15605)	*Escherichia coli EC93*	Poole *et al*., 2011
CdiA-CT^ECL^	rRNase	-	E3 rRNase (PF09000)	*Enterobacter cloacae*	Beck *et al*., 2014
CdiA-CT^EC16^	rRNase	-	E3 rRNase (PF09000)	*Dickeya chrysanthemi*	Beck *et al*., 2014
CdiA-CT^49162^	rRNase	-	E3 rRNase (PF09000)	*Enterobacter hormaechei*	Beck *et al*., 2014
CdiA-CT^0038^	rRNase	-	E3 rRNase (PF09000)	*Pseudomonas viridiflava*	Beck *et al*., 2014
**T6SS**					
ET4	DNase	His-Me_finger (CL0263)	Colicin DNase (PF12639)	*Escherichia coli PE086*	Ma *et al*., 2017
Hcp-ET1	DNase	His-Me_finger (CL0263)	HNH (PF01844)	*Escherichia coli STEC004*	Ma *et al*., 2017
RhsA	DNase	His-Me_finger (CL0263)	Endonuclea_NS_2 (PF13930)	*Dickeya dadantii*	Koskiniemi *et al*., 2013
RhsB	DNase	His-Me_finger (CL0263)	HNH (PF01844)	*Dickeya dadantii*	Koskiniemi *et al.*, 2013
Rhs2	DNase	His-Me_finger (CL0263)	HNH (PF01844)	*Serratia marcescens*	Alcoforado-Diniz and Coulthurst, 2015
Rhs2	DNase	His-Me_finger (CL0263)	AHH (PF14412)	*Acinetobacter baumannii*	Fitzsimons *et al*., 2018
TseI	DNase	His-Me_finger (CL0263)	Tox-HNH-EHHH (PF15657)	*Aeromonas dhakensis*	Pei *et al*., 2020
Tse7 (PA0099)	DNase	His-Me_finger (CL0263)	Tox-GHH2 (PF15635)	*Pseudomonas aeruginosa*	Hachani *et al*., 2014; Pissaridou *et al*., 2018
Tke2	DNase RNase	His-Me_finger (CL0263)	Colicin DNase (PF12639)	*Pseudomonas putida*	Bernal *et al*., 2017
Tke4	DNase RNase	His-Me_finger (CL0263)	Tox-SHH (PF15652)	*Pseudomonas putida*	Bernal *et al*., 2017
Txe1	DNase	His-Me_finger (CL0263)	-	*Pseudomonas plecoglossicida*	Li *et al*., 2022
Txe2	DNase	His-Me_finger (CL0263)	AHH (PF14412)	*Pseudomonas plecoglossicida*	Li *et al*., 2022
Txe4	DNase	His-Me_finger (CL0263)	Tox-SHH (PF15652)	*Pseudomonas plecoglossicida*	Li *et al*., 2022
VP1415	DNase	His-Me_finger (CL0263)	AHH (PF14412)	*Vibrio parahaemolyticus*	Salomon *et al*., 2014
Hcp-ET3	DNase	-	-	*Escherichia coli UT189*	Ma *et al*., 2017
VgrG-NucSe1	DNase	His-Me_finger (CL0263)	HNH (PF01844)	*Salmonella arizonae*	Blondel *et al*., 2009; Ho *et al*., 2017
VPA1263	DNase	His-Me_finger (CL0263)	Colicin DNase (PF12639)	*Vibrio parahaemolyticus*	Salomon *et al*., 2014; Fridman *et al*., 2022
PT1	DNase	-	-	*Escherichia marmotae*	Nachmias *et al*., 2022
IdrD	DNase	PD-(D/E)XK (CL0236)	-	*Proteus mirabilis*	Sirias *et al.*, 2020
PoNe	DNase	PD-(D/E)XK (CL0236)	-	*Vibrio parahaemolyticus*	Jana *et al*., 2019
RhsB	DNase	PD-(D/E)XK (CL0236)	-	*Acidovorax citrulli*	Pei *et al*., 2022
TseT	DNase	PD-(D/E)XK (CL0236)	Tox-REase-5 (PF15648)	*Pseudomonas aeruginosa*	Burkinshaw *et al*., 2018; Wen *et al*., 2021
TseTBg	DNase RNase	PD-(D/E)XK (CL0236)	Tox-REase-5 (PF15648)	*Burkholderia gladioli*	Yadav *et al*., 2021
TseV	DNase	PD-(D/E)XK (CL0236)	VRR_NUC (PF08774)	*Pseudomonas aeruginosa*	Wang *et al*., 2021
TseV2/TseV3	DNase	PD-(D/E)XK (CL0236)	VRR_NUC (PF08774)	*Salmonella bongori*	Hespanhol *et al*., 2022
Tce1	DNase	-	toxin_43/Ntox15 (PF15604)	*Pseudomonas putida*	Song *et al*., 2021
Tde1/2	DNase	-	toxin_43/Ntox15 (PF15604)	*Agrobacterium tumefaciens*	Ma *et al.*, 2014; Bondage *et al.*, 2016
SED_RS01930	RNase	-	Ntox47 (PF15540)	*Salmonella enterica* Dublin	Amaya *et al*., 2022
Tre23	ADP-ribosyltranferase	-	Tox-ART-HYD1 (PF15633)	*Photorhabdus laumondii*	Jurenas *et al.*, 2021
RhsP2	ADP-ribosyltranferase	-	-	*Pseudomonas aeruginosa*	Bullen *et al*., 2022
DddA	Deamination	Cytidine deaminase-like (CL0109)	DddA-like (PF14428)	*Burkholderia cenocepacia*	Mok *et al.*, 2020; Moraes *et al.,* 2021
SsdA	Deamination	Cytidine deaminase-like (CL0109)	DYW_deaminase (PF14432)	*Pseudomonas syringae*	Moraes *et al.*, 2021
**T7SS**					
EsaD/EssD	DNase	His-Me_finger (CL0263)	Endonuclea_NS_2 (PF13930)	*Staphylococcus aureus*	Cao *et al*., 2016; Ohr *et al*., 2017
YeeF	DNase	His-Me_finger (CL0263)	Endonuclea_NS_2 (PF13930)	*Bacillus subtilis*	Holberger *et al.*, 2012; Kaundal *et al.,* 2020
PT7	DNase	-	-	*Bacillus cereus BAG3X2-1*	Nachmias *et al*., 2022
YobL	rRNase	His-Me_finger (CL0263)	LHH (PF14411)	*Bacillus subtilis*	Holberger *et al*., 2012
YxiD	rRNase	His-Me_finger (CL0263)	-	*Bacillus subtilis*	Holberger *et al*., 2012
YqcG	RNase	His-Me_finger (CL0263)	GH-E (PF14410)	*Bacillus subtilis*	Holberger *et al*., 2012
BC_0920	RNase	EndoU (CL0695)	EndoU_bacteria (PF14436)	*Bacillus cereus*	Holberger *et al*., 2012
**OME**					
SitA1	DNase	His-Me_finger (CL0263)	Colicin DNase (PF12639)	*Myxococcus xanthus*	Vassallo *et al*., 2017
SitA2	DNase	His-Me_finger (CL0263)	Colicin DNase (PF12639)	*Myxococcus xanthus*	Vassallo *et al*., 2017
SitA3	tRNase	PD-(D/E)XK (CL0236)	CdiA_C (PF18451)	*Myxococcus xanthus*	Vassallo *et al*., 2017
**Nanotube**					
WapA-CT^168^	tRNase	unknown	-	*Bacillus subtilis*	Koskiniemi *et al*., 2013
WapA-CT^natto^	tRNase	unknown	-	*Bacillus subtilis*	Koskiniemi *et al*., 2013
WapA-CT^T-UB-10^	tRNase	unknown	-	*Bacillus subtilis*	Koskiniemi *et al*., 2013
WapA-CT ^PY79^	tRNase	unknown	-	*Bacillus subtilis*	Stempler *et al.*, 2017
**OMV**					
MafB^MGI-1NEM8013^	RNase	EndoU (CL0695)	EndoU_bacteria (PF14436)	*Neisseria meningitidis*	Jamet *et al*., 2015
**Antibiotic**					
Bleomycin, Phleomycin, Tallysomycin, Zorbamycin	DNase	Glycopeptides	Bleomycins	*Streptomyces verticillus*	Umezawa *et al.*, 1966; Takeshita et al., 1978; Kross *et al.*, 1982; Hecht, 2000
Calicheamicin	DNase	-	Enediynes	*Micromonospora echinospora ssp. calichensis*	Zein *et al*., 1988
Daunorubicin	DNase	-	Anthracyclines	*Streptomyces peucetius*	Marco *et al*., 1975
Kibdelomycin	DNA gyrase	-	-	*Kibdelosporangium sp. (MA7385)*	Philips *et al*., 2011
Amycolamicin	DNA gyrase	-	-	*Amycolatopsis sp. (MK575-fF)*	Sawa *et al*., 2012
Coumarin	DNA gyrase	-	-	*Streptomyces spp.*	Maxwell and Lawson, 2003; Oblak *et al.,* 2007
Cyclothialidine	DNA gyrase	-	-	*Streptomyces filipinensis NR0484*	Goetschi *et al*., 1993; Oblak *et al.,* 2007
Rifamycin	RNA polymerase	Macrolides	Ansamycin	*Amycolatopsis rifamycinica*	Sensi *et al.*, 1959; Campbell *et al.,* 2001; Floss and Yu, 2005
Fidaxomicin	RNA polymerase	Macrolides	Lipiarmycin	*Dactylosporangium aurantiacum subsp. hamdenensis*	Theriault *et al*, 1987; Artsimovitch *et al*., 2012
Gentamicin, Streptomycin, Hygromycin, Neomycin, Paromomycin, Kanamycin, Spectinomycin, Kasugamycin, Spectinomycin	16S rRNA	Aminoglycoside	-	*Actinomycetes*	Schatz *et al.*, 1944; Waksman and Lechevalier, 1949; Umezawa *et al.*, 1957; Mann and Bromer, 1958; Mason *et al.*, 1961; Weinstein *et al.*, 1963; Wilson, 2009
Tetracycline	16S rRNA	Tetracyclines	-	*Streptomyces aureofaciens*	Putnam *et al.*, 1953; Brodersen *et al.,* 2000; Pioletti *et al.,* 2001
Pactamycin	16S rRNA	-	Aminocyclopentitol	*Streptomyces pactum*	Bhuyan, 1962; Brodersen *et al.,* 2000
Edeine	16S rRNA	-	Edeine	*Brevibacillus brevis*	Kurylo-Borowska, 1959; Pioletti *et al.,* 2001
Erythromycin	23S rRNA	Macrolides	-	*Actinomycetes*	Schlünzen *et al.,* 2001; Reviewed by Vázquez-Laslop and Mankin, 2018;
Lincomycin	23S rRNA	-	Lincosamides	*Streptomyces lincolnensis*	Mason *et al.*, 1962
Blasticidin S	23S rRNA	-	Aminoacyl nucleoside	*Streptomyces griseochromogenes*	Takeuchi *et al*., 1958; Hansen *et al.,* 2003
Viomycin/Capreomycin	16S rRNA 23S rRNA	Cyclic peptides	Tuberactinomycins	*Streptomyces puniceus*	Finlay *et al*., 1951; Herr and Redstone, 1966; Johansen *et al*, 2006

## His-Me finger superfamily

The most representative superfamily of DNases is the His-Me finger, also known as HNH superfamily, named after the first characterized enzyme showing the conserved His-Asn-His residues (Wu *et al*., 2020). This superfamily is defined by the compact catalytic conserved ββα-fold, consisting of a β-hairpin followed by an α-helix in which a highly conserved histidine (H) is located at the end of the first β-strand and a metal-binding conserved residue in α-helix (Zn^2+^ or Mg^2+^) (Wu *et al*., 2020), thus the name His-Me finger. His-Me finger is thought to mediate nonspecific DNA cleavage, with the α-helix fitting into the DNA minor groove, which aligns the β-hairpin with the DNA phosphodiester backbone (Flick *et al*., 1998). For cleavage, the metal ion destabilizes the scissile phosphodiester and neutralize the negatively charged transition state (Maté and Kleanthous, 2004). The conserved H residue then activates a water molecule for a nucleophilic attack on the scissile phosphate to hydrolyze the bond (Yang *et al*., 2011). Even though the amino acid sequences of members of this superfamily are incredible variable, the compact ββα-fold and catalytic mechanism is well conserved (Jablonska *et al*., 2017; Wu *et al*., 2020). This fold is present in all kingdoms of life, and in bacteria the enzymes have variable functions spanning from genome maintenance to host defense and target offense (Wu *et al*., 2020).

All the characterized His-Me finger bacteriocins and effectors described to date that were empirically tested were shown to degrade genomic or plasmid DNA in a nonspecific manner (Table 1, Figure 2). These include several colicins from *E*. *coli* (Schaller and Nomura, 1976; Males and Stocker, 1980; Cooper and James, 1984; Toba *et al*., 1988; Chak *et al*., 1991; Chak *et al*., 1996; Garinot-Schneider *et al*., 1996; Pommer *et al*., 1998; Kurazono *et al*., 2000; Hsia *et al*., 2004; Nipič *et al*., 2013; Zaw *et al*., 2013). Other bacteria also encode bacteriocins from the His-Me superfamily, such as *Pseudomonas aeruginosa* (Ohkawa *et al*., 1973; Sano and Kageyama, 1981; Sano *et al*., 1993; Ghequire and De Mot, 2014; Turano *et al*., 2017; Turano *et al*., 2020), *Klebsiella pneumoniae* (Cooper and James, 1985; James *et al*., 1987; Riley *et al*., 2001) and *Pectobacterium carotovorum* (Roh *et al*., 2010) (Table 1). 

Moreover, there are secreted effectors belonging to the His-Me superfamily (Table 1) such as T6SS effectors RhsA (rearrangement hotspot A) and RhsB from *Dickeya dadantii* (Koskiniemi *et al*., 2013). These Rhs effetors were shown to confer competitive advantage to *D*. *dadantii*, inducing loss of DAPI (4′,6-diamidino-2-phenylindole) staining in target cells and leading to plasmid degradation in overexpressing bacteria (Koskiniemi *et al*., 2013). Other organisms that encode T6SS effectors from the His-Me superfamily are *Vibrio parahaemolyticus* (Salomon *et al*., 2014; Fridman *et al*., 2022), *Serratia marcescens* (Alcoforado-Diniz and Coulthurst, 2015), *Acinetobacter baumannii* (Fitzsimons *et al*., 2018), *E*. *coli* (Nipič *et al*., 2013; Ma *et al*., 2017), *Aeromonas dhakensis* (Pei *et al*., 2020), and *Pseudomonas spp*. (Hachani *et al*., 2014; Bernal *et al*., 2017; Pissaridou *et al*., 2018; Li *et al*., 2022). In addition, the T7SS effectors EsaD (Ess-associated gene D) from *Staphylococcus aureus* (Cao *et al*., 2016; Ohr *et al*., 2017) and YeeF-CT from *Bacillus subtilis* (Holberger *et al*., 2012; Kaundal *et al*., 2020) are representatives from this superfamily. Predicted T4SS effectors also encode nuclease domains, such as Smlt4382 from *Stenotrophomonas maltophilia* (Bayer-Santos *et al*., 2019) and XAC3266 from *Xanthomonas citri* (Souza *et al*., 2015), both with AHH domain (PF14412). Besides protein secretion systems, SitA1 and SitA2 toxins from *Myxococcus xanthus* also belong to the His-Me superfamily and are delivered via outer membrane exchange events in which bacteria donate and receive outer membrane material from kin (Vassallo *et al*., 2017). The extensive list of DNases containing the conserved ββα-fold demonstrate that it is widely distributed weapon used during antagonistic interactions.

Although most toxins belonging to the His-Me finger target DNA, there are a few examples that also target RNA. Examples include colicin E7, which shows both DNase and RNase activity *in vitro* (Hsia *et al*., 2004), and colicin E9 (Pommer *et al*., 2001). Moreover, there are two effectors that have been shown to exclusively target RNA: YobL and YxiD from the T7SS of *B*. *subtilis* cleave rRNA *in vivo* (Holberger *et al*., 2012). These demonstrade the versatility of domains with the ββα-fold to cleave different subtrates.

It is worth highlighting that most effectors described above are polymorphic toxins, harboring a translocation N-terminal domain in addition to their toxic C-terminal domains. These include RhsA and RhsB with a N-terminal RHS_repeat (PF05593) and C-terminal Endonuc_NS_2 (PF13930) and HNH domains (PF01844), respectively (Koskiniemi *et al*., 2013); VP1415 with N-terminal PAAR (PF05488) and C-terminal AHH (PF14412) domains (Salomon *et al*., 2014); Hcp-ET1 with N-terminal Hcp (PF05638) and C-terminal HNH domains (Ma *et al*., 2017); SARI_02603 with N-terminal VgrG (PF04717) and C-terminal HNH (PF01844) domains (Blondel *et al*., 2009; Ho *et al*., 2017).

## PD-(D/E)xK superfamily

The PD(D/E)xK superfamily is the second most abundant among bacteriocins and effectors with nuclease activity. Like the His-Me finger, proteins belonging to PD(D/E)xK share small amino acid sequence similarity, but present conserved secondary structure signatures (Steczkiewicz *et al*., 2012). The conserved fold of this group comprise an α-helix followed by three antiparallel β-strands and a second α-helix followed by a final β-strand (αβββαβ) (Steczkiewicz *et al*., 2012). The catalytic residues are located in the second and third β-strand; the first α-helix has structural role and is related to the formation of the active site, while the second α-helix is involved in substrate binding (Wah *et al*., 1998). Conserved aspartic acid and glutamic acid (D/E) residues coordinate the metal ion (usually Mg^2+^), while the conserved lysine (K) associates with a water molecule to hydrolyze the phosphodiester bond (Kelly *et al*., 2007). This superfamily includes enzymes related to DNA metabolism (Steczkiewicz *et al*., 2012).

Effectors belonging to the PD(D/E)xK superfamily degrade both DNA and RNA (Table 1, Figure 2). These include T5SS effectors CdiA-CT_II_^Bp1026b^ and CdiA-CT^E479^ from *Burkholderia pseudomallei* (Morse *et al*., 2012; Nikolakakis *et al*., 2012), and CdiA_2_-CT from *A*. *baumannii* (Roussin *et al*., 2019). Examples of T6SS effectors belonging to the PD(D/E)xK are TseT from *P*. *aeruginosa* (Burkinshaw *et al*., 2018; Wen *et al*., 2021), TseTBg from *Burkholderia gladioli* (Yadav *et al*., 2021), PoNe (polymorphic nuclease effector) from *V*. *parahaemolyticus* (Jana *et al*., 2019), IdrD from *Proteus mirabilis* (Sirias *et al*., 2020), RhsB from *Acidovorax citrulli* (Pei *et al*., 2022), and TseV from *P*. *aeruginosa* and *Salmonella bongori* (Wang *et al*., 2021; Hespanhol *et al*., 2022). In addition, SitA3 involved in interbacterial antagonism via outer membrane exchange contains the conserved αβββαβ fold (Vassallo *et al*., 2017).

The first PD-(D/E)xK effector was described in *P*. *aeruginosa* (TseT) and contains a Tox-REase-5 domain (PF15648) (Zhang *et al*., 2012; Burkinshaw *et al*., 2018). Homologs of TseT have been characterized in *B*. *gladioli* (TseTBg1 and TseTBg2) (Yadav *et al*., 2021), and degrade both DNA and RNA (Yadav *et al*., 2021). Interestingly, the DNase activity of TseTBg was affected by methylation. A DNA methylase (Dam^BG^) is encoded next to the effector, and plasmids isolated from Dam^BG^-producing *E*. *coli* were not degraded by TseTBg1 or TseTBg2 (Yadav *et al*., 2021). In addition, point mutations in conserved aspartic acid (D) and lysine (K) of TseTBg1 and TseTBg2 abrogated DNase activity (Yadav *et al*., 2021). Another curiosity is that these effectors are encoded next to two cognate immunity proteins: one of them neutralizes the enzymatic activity *in vitro* while the second directly binds to the promoter region of the effector, acting as a transcriptional repressor (Yadav *et al*., 2021).

The VRR-Nuc (virus-type replication repair nuclease) domain is found in enzymes involved in interstrand DNA crosslink repair (Kratz *et al*., 2010; Liu *et al*., 2010; MacKay *et al*., 2010; Smogorzewska *et al*., 2010; Gwon *et al*., 2014; Wang *et al*., 2014; Zhao *et al*., 2014), but recent studies identified effectors containing this domain - named TseVs (type VI effector VRR-Nuc) (Wang *et al*., 2021; Hespanhol *et al*., 2022). TseV2 and TseV3 from *S*. *bongori* were shown to participate in interbacterial competition in a T6SS-dependent manner (Hespanhol *et al*., 2022). TseV3 is a structure-specific nuclease that cleaves DNA substrates with a Y shape (named splayed arm), which resemble replication forks or transcription bubbles (Hespanhol *et al*., 2022). TseV2 and TseV3 induce DNA double-strand breaks and activate the SOS response *in vivo* (Hespanhol *et al*., 2022).

Enzymatic assays also showed the ability of additional PD-(D/E)xK superfamily members to degrade DNA *in vitro*. These include PoNe (Jana *et al*., 2019), RhsB (Pei *et al*., 2022), and IdrD (Sirias *et al*., 2020). Moreover, the T5SS effectors CdiA_2_-CT^Ab30011^ from *A*. *baumannii* (Roussin *et al*., 2019) and CdiA-CT^E479^ from *B*. *pseudomallei* (Nikolakakis *et al*., 2012) were experimentally shown to degrade nucleic acids, leading to cell growth arrest. The first induces target cell DNA damage, while the second is specific to tRNA^Arg^ (Nikolakakis *et al*., 2012; Roussin *et al*., 2019). In summary, similar to the His-Me finger representatives, PD(D/E)xK members can target both DNA and RNA molecules.

## E3-rRNase family

Members of the E3 rRNase family (PF09000) are the most frequent found in bacteriocins and effectors that target ribosomal RNAs (Table 1, Figure 2). Colicin E3 from *E*. *coli* was the first to be characterized (Bowman *et al*., 1971; Senior and Holland, 1971; Lasater *et al*., 1989; Ogawa *et al*., 1999), hence the name of the group E3-rRNase. Several homologs were later identified, such as colicin E4 and E6 from *E*. *coli* (Horak, 1975; Males and Stocker, 1982; Šmarda *et al*., 1988; Sharma *et al*., 2002; Hirao *et al*., 2004), cloacin DF13 from *Enterobacter cloacae* (De Graaf *et al*., 1973), pyocin S6 from *P*. *aeruginosa* (Dingemans *et al*., 2016) and klebicin C from *K*. *pneumoniae* (Chavan *et al*., 2005). The E3-rRNase domain has a highly specific activity towards the phosphodiester bond between nucleotides adenine_1493_ and guanine_1494_ of the 16S rRNA (Lasater *et al*., 1989). The T5SS effectors CdiA-CT^ECL^ from *E*. *cloacae* and CdiA-CT^EC16^ from *Erwinia chrysanthemi* contain an E3-rRNase domain and display activity against the 16S rRNA at the same position (Beck *et al*., 2014). CdiA-CT^49162^ and CdiA-CT^0038^ from *Enterobacter hormaechei* and *Pseudomonas viridiflava* are homologs that contain the E3-rRNase domain; however, their enzymatic activity was not experimentally validated (Beck *et al*., 2014).

## Colicin D/E5 superfamily

The first member of the Colicin D/E5 clan (CL0640) was isolated from *E*. *coli* and named colicin D (Timmis and Hedges, 1972). Later, a second member of this clan was identified in *Shigella sonnei* and called colicin E5 (Males and Stocker, 1982). This protein is homologous to colicin E3 in the receptor-binding and translocation domains but shows a distinct toxic domain (Yajima *et al*., 2006). Both colicin E5 and colicin D were shown to be ribonucleases that target tRNAs and cleave anticodon loops between the 34 and 35 nucleotides of queuine-containing tRNAs, and between the 38 and 39 nucleotides of tRNAs^Arg^, respectively (Ogawa *et al*., 1999; Tomita *et al*., 2000; Masaki and Ogawa, 2002). The catalytic domain found in these colicins were grouped with other metal-independent RNases as part of the BECR-fold (Barnase-EndoU-ColicinD/E5-RelE), which contain a similar structure composed of a α-helix and an anti-parallel β-sheet formed by four strands (Zhang *et al*., 2012). In colicin D, a large positively charged surface promotes tRNA binding and brings the anticodon loop close to a histidine residue located at the α-helix (His_611_), which carries the catalytic function by acting as a general base (Yajima *et al*., 2004). Colicin E5 possesses a positively charged cleft that promotes RNA docking (Lin *et al*., 2005) and targets tRNA^His^, tRNA^Tyr^, tRNA^Asn^ and tRNA^Asp^ between their modified queuine nucleotide Q34 and U35 (Ogawa *et al*., 1999). The catalytic residues that participate in E5 enzymatic activity do not include a catalytic histidine that usually participate in RNA cleavage (Lin *et al*., 2005; Yajima *et al*., 2006), but instead residues R33 and K25 act as acid-base pairs (Inoue-Ito *et al*., 2012).

Besides colicin D and E5, other bacterial effectors have been described to belong to this clan (Table 1, Figure 2). Pyocin S4 from *P*. *aeruginosa* (Parret and De Mot, 2000) and klebicin D from *K*. *pneumoniae* (Chavan *et al*., 2005) have C-terminal domains that belong to the colicin D/E5 superfamily, and carocin S2 from *P*. *carotovorum* has ribonuclease activity *in vitro* (Chan *et al*., 2011). The CDI system has a variety of effectors that belong to this clan. The CdiA-CT^EC869^ and CdiA-CT^EC3006^ from *E*. *coli* are tRNases that have a different cleavage site located at the tRNA acceptor stem (Willett *et al*., 2015; Jones *et al*., 2017; Gucinski *et al*., 2019), the same is observed for CdiA-CT^Kp342^ from *K*. *pneumoniae* (Gucinski *et al*., 2019). CdiA-CT^K96243^ and CdiA-CT^E478^ from *B*. *pseudomallei* present the same activity as colicin E5 (Aoki *et al*., 2010; Nikolakakis *et al*., 2012). In summary, members of the colicin D/E5 superfamily target tRNA by cleaving at distinct sites.

## EndoU superfamily

EndoU RNases comprise nucleases from eukaryotic and viral RNA-processing enzymes (Zhang *et al*., 2011) and polymorphic bacterial toxins (Zhang *et al*., 2012). As the letter “E” in the BECR fold, EndoU toxins are metal-independent ribonucleases that contain the typical four stranded β-sheet next to a α-helix structure (Zhang *et al*., 2012), and are predicted to have ribonuclease activity carried out by two histidine residues (Zhang *et al*., 2011; Michalska *et al*., 2018). This superfamily has been described to be related to Ribonuclease A (Mushegian *et al*., 2020).

Four EndoU antibacterial toxins were verified experimentally, and the results showed that this fold presents some diversity in its mode of action. The T7SS effector BC_0920 from *Bacillus cereus* has RNase activity (Holberger *et al*., 2012). MafB^MGI-1NEM8013^, an outer membrane exported toxin from *Neisseria meningitidis*, is a nonspecific ribonuclease with a preference for urydilates (Jamet *et al*., 2015). CdiA-CT^STECO31^, a T5SS secreted toxin from *E*. *Coli* (Michalska *et al*., 2018), presents a specific cleavage site at the anticodon loop of tRNA^Glu^; while CdiA-CT^GN05224^ from *Klebsiella aerogenes* shows tRNase activity *in vivo* (Michalska *et al*., 2018). 

Even though bioinformatic analysis can broadly predict protein function, the precise mode of action of each nuclease within a superfamily requires empirical biochemical assays to accurately determine activity.

## Other nuclease domains

Besides the nuclease groups mentioned above, other domains can be found in bacteriocins and effectors. Tde1 and Tde2 (type VI DNase effectors) from *Agrobacterium tumefaciens* have a Ntox15 domain (Zhang *et al*., 2012; Bondage *et al*., 2016), which is a polymorphic toxic domain characterized by an all α-helical fold and conserved HxxD catalytic residues (Zhang *et al*., 2012). Both effectors display DNase activity (Bondage *et al*., 2016). Several WapA proteins from *B*. *subtilis* display tRNAse activity, such as WapA-CT^168^, WapA-CT^natto^ and WapA-CT^T-UB-10^; however, the toxic domains remain undetermined (Koskiniemi *et al*., 2013). In addition, Wap-CT^PY79^ was hypothesized to display tRNAse activity based on sequence similarity (Stempler *et al*., 2017).

A recently discovered effector with no detectable domain and DNase activity is Tce1 (T6SS contact-independent antibacterial effector 1) from *Yersinia pseudotuberculosis* (Song *et al*., 2021). Tce1 is a Ca^2+^- and Mg^2+^-dependent enzyme that displays an interesting mechanism of target-cell delivery, which can be either dependent or independent of contact (via the outer membrane receptors BtuB and OmpF) (Song *et al*., 2021).

Also recently, new polymorfic toxin C-teminal domains (PTs) were described (Nachmias *et al*., 2022). The toxic domains of PT1 and PT7 were shown to be non-specific DNases that did not show sequence or structural similarity to any known nuclease (Nachmias *et al*., 2022). PT1 is likely secreted by the T6SS, while PT7 is probably secreted via the T7SS (Nachmias *et al*., 2022).

Other toxins with undetectable domains but with experimentally characterized nuclease activities comprise carocin S1 and S3 from *P*. *carotovorum* (Chuang *et al*., 2007; Wang *et al*., 2020), pyocin S3 from *P*. *aeruginosa* (Duport *et al*., 1995), and the T6SS effector Hcp-ET3 from *E*. *coli* (Ma *et al*., 2017). The characterization of these and other new toxic domains is an interesting source of information to the discovery of novel enzymatic activities.

## Deaminases

Deaminases are enzymes that induce the deamination of nucleotides and are related to salvage pathways of purines and pyrimidines (Nygaard, 1993). Several deaminase domains have been predicted in polymorphic toxins (Iyer *et al*., 2011; Zhang *et al*., 2012). The first characterized T6SS deaminase effector was DddA (dsDNA deaminase toxin A) from *Burkholderia cenocepacia* (Mok *et al*., 2020). DddA promotes deamination of cytosine and its conversion to uracil in dsDNA, leading to a DNA mismatch during replication that needs to be repaired by the base excision repair (BER) pathway (Uphoff and Sherratt, 2017; de Moraes *et al*., 2021). An example of deaminases targeting ssDNA is the T6SS effector SsdA (ssDNA deaminase toxin A) from *Pseudomonas syringae*, which deaminases cytosine into uracil (de Moraes *et al*., 2021). Sublethal doses of DddA are related to an increase in the frequency of mutations, with a preference for C/G to A/T substitutions (Mok *et al*., 2020; de Moraes *et al*., 2021). The action of these mutagenic effectors can promote antibiotic resistance in natural settings (de Moraes *et al*., 2021).

## ADP-ribosyltransferases

ADP-ribosyltranferases (ARTs) are enzymes able of transferring an ADP-ribose from the cofactor β-nicotinamide adenine dinucleotide (NAD^+^) into certain targets, which could be either amino acids or nucleotides (Mikolčević *et al*., 2021). In bacteria, many ARTs are virulence factors involved in pathogenesis that modify specific host cell proteins to manipulate cellular functions (Yoshida and Tsuge, 2021). These ARTs can be classified into two families: diphtheria toxin (DTX) with the conserved residues H-Y-E, and cholera toxin (CTX) with the conserved residues R-S-E (Mikolčević *et al*., 2021). 

Among the weapons used in interbacterial antagonism, Tre23 (type VI secretion ADP-ribosyltranferase effector 23) from *Photorhabdus laumondii* is an ART from the H-Y-E clade that transfers ADP-ribose to 23S rRNA (Jurėnas *et al*., 2021). This modification occurs at the 23S rRNA GTPase-associated site of the ribosome, which is necessary for elongation during translation, thus stopping protein synthesis (Jurėnas *et al*., 2021) (Figure 2). Another RNA modifying toxin is RhsP2 from *P*. *aeruginosa* (Bullen *et al*., 2022). Interestingly, this enzyme displays the conserved residues Y-E and E from the two DTX and CTX ART families (Bullen *et al*., 2022). RhsP2 ADP-ribosylates a series of non-coding RNAs in target cells, including 4.5S rRNA, 6S rRNA, tRNAs, hindering multiple essential pathways (Bullen *et al*., 2022). Thus, ART toxins provide another layer of antagonistic strategies that bacteria use to interfere with molecules of the central dogma.

## Antibacterial Small Molecules Targeting Nucleic Acids

Bacteria produce several classes of antibiotics that target nucleic acids, such as aminoglycosides, tetracyclines and macrolides (Table 1, Figure 2). The structural diversity of these molecules provides distinct opportunities for inhibition of the information flow thought the central dogma. Some antibiotics can induce DNA cleavage, inhibit DNA gyrases/topoisomerases or RNA polymerases, or bind to ribosomal RNAs to interfere with protein synthesis. 

Among the antibiotics that induce DNA cleavage there are bleomycins, calicheamicin and daunorubicin. The bleomycin group comprises bleomycins, phleomycins, tallysomycin and zorbamycins (Hecht, 2000). Bleomycins are glycopeptides first isolated from *Streptomyces verticillus* (Umezawa *et al*., 1966) that promote oxidative cleavage of double-strand DNA in a sequence-specific manner (Takeshita *et al*., 1978; Kross *et al*., 1982). These antibiotics rely on the presence of molecular oxygen and a redox active metal like Fe^2+^ or Cu^+^ (Burger *et al*., 1981; Hecht, 2000). Bleomycins are composed of four functional domains: metal-binding, DNA-binding, linker region connecting the two previous domains, and a disaccharide moiety that promotes cell selectivity (Boger and Cai, 1999). The metal-binding domain is responsible for the specificity of DNA sequence (Sugiyama *et al*., 1986), which consists mainly of GT dinucleotides but can also be GC and AT (Kross *et al*., 1982). Phleomycins, tallysomycins and zorbamycins have slightly different sequence specificity but cleave DNA in a similar mechanism (Kross *et al*., 1982). Calicheamicin belongs to the enediynes group of antibiotics and was first isolated from *Micromonospora echinospora ssp*. *calichensis* (Zein *et al*., 1988). It promotes double-strand DNA cleavage in a sequence-specific manner, preferentially at AGGA, TCCT and ACCT (Zein *et al*., 1988). The mechanism of cleavage requires the removal of hydrogen atoms (abstraction) from the DNA backbone (Lee *et al*., 1991). Daunomycin from *Streptomyces peucetius* can intercalate and form complexes with DNA, leading to chromosome fragmentation (Marco *et al*., 1975).

Some antibiotics promote DNA degradation by arresting topoisomerases. Type II topoisomerases function by promoting metal-dependent DNA double-strand breaks, followed by ATP-dependent translocation of DNA segments and rejoining the separated DNA ends (Gentry and Osheroff, 2013). The DNA gyrase and topoisomerase IV (topo IV) are type II topoisomerases found in bacteria and are composed of two domains: GyrA and GyrB, and ParC and ParE, respectively (Levine *et al*., 1998). The GyrA or ParC domains interact with DNA, while GyrB or ParE bind and hydrolyze the ATP necessary for enzymatic function (Levine *et al*., 1998). Some groups of antibiotics bind to the ATP-binding site of GyrB and ParE to inhibit the activity of the topoisomerase complex, thus generating DNA breaks and the collapse of the replication fork (Anderson *et al*., 2000; Maxwell and Lawson, 2003). These antibiotics comprise coumarins and cyclothialidines from *Streptomyces spp*. (Goetschi *et al*., 1993; Oblak *et al*., 2007), kibdelomycin from *Kibdelosporangium sp*. (Phillips *et al*., 2011), and amycolamicin from *Amycolatopsis sp*. (Sawa *et al*., 2012).

Transcription is another seductive target for antibacterial natural products. Rifamycin from *Amycolatopsis rifamycinica* (Sensi, 1959) is a macrolide antibiotic that blocks transcription by binding to the β subunit of the RNA polymerase, thus stopping DNA-dependent RNA synthesis via transcript elongation arrest (Campbell *et al*., 2001; Floss and Yu, 2005). Fidaxomicin isolated from *Dactylosporangium aurantiacum* (Theriault *et al*., 1987) prevents RNA transcription by blocking DNA double-strand opening in promotor regions, thus inhibiting transcription initiation by the RNA polymerase (Artsimovitch *et al*., 2012).

The ribosome is the center of protein synthesis. It is a large ribonucleoprotein complex composed of two subunits (30S and 50S) forming the 70S bacterial ribosome. The 30S subunit contain the 16S rRNA, while the 50S subunit contain the 23S rRNA and 5S rRNA (Deutscher, 2009). These nanomachines are one of the favorite targets when it comes to bacterial growth inhibition by antibiotics. Most of these antibacterial molecules inhibit ribosome activity by binding directly to the rRNAs and arresting translation by acting as allosteric inhibitors. Here we focused only on antibiotics produced by bacteria that interfere with protein synthesis by binding to rRNAs.

The 30S ribosomal subunit is the target of aminoglycosides, tetracyclines, pactamycin and edeine, which bind at different sites of the 16S rRNA. Aminoglycosides gentamicin from *Micromonospora spp*. (Weinstein *et al*., 1963), hygromycin B from *Streptomyces hygroscopicus* (Mann and Bromer, 1958), neomycin from *Streptomyces fradiae* (Waksman and Lechevalier, 1949), paromomycin from *Streptomyces krestomuceticus*, kanamycin from *Streptomyces kanamyceticus* (Umezawa *et al*., 1957) and streptomycin from *Streptomyces griseus* (Schatz *et al*., 1944) can target the helix 44 of 16S rRNA (Wilson, 2009). Meanwhile, aminoglycoside spectinomycin from *Streptomyces spectabilis* (Mason *et al*., 1961) targets the helix 34 of 16S rRNA (Wilson, 2009). Lastly, aminoglycoside kasugamycin from *Streptomyces kasugaensis* (Umezawa *et al*., 1965) binds to 16S rRNA at the messenger RNA channel (Schuwirth *et al*., 2006). Tetracycline from *Streptomyces aureofaciens* (Putnam *et al*., 1953) binds to helixes 31 and 34 (Brodersen *et al*., 2000; Pioletti *et al*., 2001). Pactamycins from *Streptomyces pactum* (Bhuyan, 1962) binds at the central domain of 16S rRNA (Brodersen *et al*., 2000), while edeine from *Brevibacillus brevis* (Kurylo-borowska, 1959) binds to helixes 44 and 45 (Pioletti *et al*., 2001).

The 50S subunit is also widely affected by antibiotics. Erythromycin, lincomyicin, blasticidin, viomycin and capreomycin target the 23S rRNA. Antibiotics from the macrolide class are produced by diverse Actinomycetes (Dinos, 2017) and can bind to the 23S rRNA at the nascent peptide exit tunnel (Schlünzen *et al*., 2001; Vázquez-Laslop and Mankin, 2018). Lincomycin from *Streptomyces lincolnensis* (Mason *et al*., 1962) binds to the peptidyl transferase cavity at the ribosomal A site (Douthwaite, 1992). Blasticidin S from *Streptomyces griseo chromogenes* (Takeuchi *et al*., 1958) and sparsomycin from *Streptomyces sparsogenes* (Owen *et al*., 1962) bind to the 23S rRNA at the ribosomal P site (Johnston *et al*., 2002; Hansen *et al*., 2003). Tuberactinomycins, such as capreomycin from *Streptomyces capreolus* (Herr Jr and Redstone, 1966) and viomycin from *Streptomyces puniceus* (Finlay *et al*., 1951), can interact with both 30S and 50S ribosomal subunits by binding to 16S rRNA at helix 44 and to 23S rRNA at helix 69 (Johansen *et al*., 2006). In summary, antibiotics collectively work in several steps to prevent the information flow through the central dogma.

## Contribution to the Development of Antibiotic Resistance

During the evolutionary arms race in which bacteria developed several weapons to inactivate or kill competitors, immunity mechanisms to prevent self-intoxication and protect sister-cells evolved concomitantly. For proteinaceous antibacterial molecules like effectors and bacteriocins, the expression of a specific immunity protein is usually the most common mechanism of defense (Zhang *et al*., 2012; Ruhe *et al*., 2020). For small molecules like antibiotics, there are several mechanisms that could render a cell resistant: (1) target modification by specific enzymes; (2) target bypass via mutations in the targets that lead to reduced affinity; (3) degrading or modifying proteins that act on the molecules; (4) reduced intake via altered membrane permeability; (5) efflux pumps that export the molecules (Darby *et al*., 2022).

During interbacterial competitions, effectors and bacteriocins that target the DNA contribute to the emergence of antibiotic resistance by increasing the rate of mutagenesis in cells that receive a sublethal dose. The deaminase T6SS effector DddA has been shown to increase the rate of C/G to T/A mutation, leading to emergence of rifamycin resistance by introducing point mutations in the *rpoB* gene, which encodes the β-subunit of RNA polymerase (de Moraes *et al*., 2021). In addition, cleavage of the 16S rRNA by colicin E3 promotes faster tRNA-mRNA translocation in ribosomes, thus making it less sensitive to inhibition by the antibiotic viomycin (Lancaster *et al*., 2008).

In general, DNA damage induced by bacteriocins or effectors activate the SOS response, which can induce the activation of the translesion DNA repair pathway and promote mutations (Patel *et al*., 2010). The mutagenesis can also be responsible for altering gene expression or characteristics of membrane channels important for antibiotic internalization (Livermore, 1990). Mutations in the promoter region of OmpF (outer membrane protein F) leads to its downregulation, thus conferring β-lactam resistance in *E*. *coli* (Delcour, 2009). Similarly, point mutations in OmpF in *Enterobacter aerogenes* reduce outer membrane permeability and promote resistance to β-lactam antibiotics, which act by inhibiting peptidoglycan synthesis (Dé *et al*., 2001).

In addition to contributing to an increase in the mutation rate of target cells, antibacterial molecules (e.g., lipases and peptidoglycan hydrolases) can promote the lysis of target cells and the release of extracellular DNA, which could be uptaken by the attacker bacterium and incorporated into its genome, thus stimulating horizontal gene transfer and the spread of genes encoding antibiotic resistance. Examples of this include the T6SSs of *Vibrio cholerae* and *Acinetobacter baylyi* (Borgeaud *et al*., 2015; Cooper *et al*., 2017; Ringel *et al*., 2017). Curiously, *V*. *cholerae* have its T6SS gene cluster under the control of competence regulators (Borgeaud *et al*., 2015), demonstrating the relationship between the bacterial competition and horizontal gene transfer events.

## Perspectives

Nucleases are possibly the most ancient biological weapons and likely used in periods prior to the development of individual cells surrounded by membranes. Their activities are among the chemical armaments used in biological conflicts across all organizational levels. For example, endonuclease domains of the His-Me superfamily are found in nucleic acid-degrading snake toxins, bacterial polymorphic toxins, bacterial restriction-modification systems conferring antiviral immunity, and eukaryotic apoptosis systems (Zhang *et al*., 2012; Trummal *et al*., 2014; Jablonska *et al*., 2017). There is still a wide array of predicted nucleic acids-targeting enzymes that require further empiral characterization. While it is possible to extropolate the possible activities of predicted groups based on similarities to known enzymes, such as Ntox18, Ntox19, Ntox22 and Ntox30 that are expected to be metal-independent RNases (Zhang *et al*., 2012), there are Ntox groups for which the nature of catalysis could not be predicted (Zhang *et al*., 2012).

The large number of antibacterial molecules targeting the central dogma and the number of resistance mechanisms promoting immunity to these molecules, call our attention to the fact that antibiotic resistance is an ancient and naturally occurring phenomenon widespread in the environment. It is important to note that these molecules attacking the central dogma act as part of a miscellaneous arsenal of toxins that damage other cellular components and their combined effect dictates the aftermath of antagonistic interactions. Experimental data confirmed that antibiotic resistance can arise solely by competitive interactions between bacteria without previous antibiotic exposure (Koch *et al*., 2014). Bacteria joined an arms race millions of years prior to the discovery of antibiotics and studying the mechanisms and outcomes of antagonistic interaction might help us anticipate the emergence of antibiotic resistance in different settings. 
